# Melanoma Prevention: Comparison of Different Screening Methods for the Selection of a High Risk Population

**DOI:** 10.3390/ijerph18041953

**Published:** 2021-02-17

**Authors:** Nevio Dubbini, Antonella Puddu, Grazia Salimbeni, Stefano Malloggi, Daniele Gandini, Pietro Massei, Giuseppe Ferraùto, Tommaso Rubino, Laura Ricci, Giovanni Menchini, Marco Celli, Maurizia Ghilardi, Roberto Gianfaldoni, Serena Gianfaldoni, Andrea Nannipieri, Antonella Romanini

**Affiliations:** 1 Miningful Studio s.r.l.s, 56127 Pisa, Italy; 2 Plastic Surgery Department, San Rossore Clinic, 56122 Pisa, Italy; pudduanto@gmail.com (A.P.); graziasalimbeni@gmail.com (G.S.); info@malloggichirurgia.it (S.M.); 3 Plastic and Reconstructive Surgery Specialist, 56122 Pisa, Italy; posta@danielegandini.it; 4 Private Plastic Surgeon, Check-Up Medical Center, 55100 Lucca, Italy; info@pietromassei.it; 5 Private Dermatologist, San Rossore Clinic, 56122 Pisa, Italy; ferrauto.dott.giuseppe@gmail.com; 6 Private Dermatologist, 56127 Pisa, Italy; rubinotommaso@tiscali.it (T.R.); menchini.g@gmail.com (G.M.); maurizia.ghilardi@gmail.com (M.G.); 7 Dermatology Department, Azienda USL Toscana Nord Ovest, 57025 Piombino, Italy; francolrt41@gmail.com; 8 Dermatology Department, Ospedale Unico Della Versilia, Lido di Camaiore, 55041 Lucca, Italy; cellimbm@tiscali.it; 9 Dermatology Department, University “G. Marconi of Rome”, 00193 Roma, Italy; robertogianfaldoni@gmail.com (R.G.); serena.gianfaldoni@gmail.com (S.G.); 10 Dermatology Department, Azienda Ospedaliero-Universitaria Pisana, 56126 Pisa, Italy; nannipieri4@hotmail.com; 11 Medical Oncology Department, Azienda Ospedaliero-Universitaria Pisana, 56126 Pisa, Italy; amvromanini@gmail.com

**Keywords:** melanoma, secondary prevention, screening

## Abstract

Background: Guidelines recommend limiting melanoma screening in a population with known risk factors, but none indicates methods for efficient recruitment. The purpose of this study is to compare three different methods of recruiting subjects to be screened for melanoma to detect which, if any, is the most efficient. Methods: From 2010 to 2019, subjects were recruited as follows: (1) regular skin examinations (RS), mainly conducted through the Associazione Contro il Melanoma network; (2) occasional melanoma screening (OS), during annual public campaigns; (3) and selective screening (SS), where people were invited to undergo a skin check after filling in a risk evaluation questionnaire, in cases where the assigned outcome was intermediate/high risk. Melanoma risk factors were compared across different screening methods. Generalized Linear Mixed Models were used for multivariable analysis. Results: A total of 2238 subjects (62.7% women) were recruited, median age 44 years (2–85), and 1094 (48.9 %) records were collected through RS, 826 (36.9 %) through OS, and 318 (14.2 %) through SS. A total of 131 suspicious non-melanoma skin cancers were clinically diagnosed, 20 pathologically confirmed, and 2 melanomas detected. SS performed significantly better at selecting subjects with a family history of melanoma and I-II phototypes compared to OS. Conclusions: Prior evaluation of melanoma known risk factors allowed for effective selection of a population to screen at higher risk of developing a melanoma.

## 1. Introduction

The incidence of malignant melanoma varies from 0.003% to 0.005% per year in Mediterranean countries, and from 0.012% to 0.020% per year in Northern European countries [1]. Melanoma age-adjusted overall incidence per 100,000 person-years has increased in the past 30 years, from 13.94 to 21.87 from 1989–1991 to 2007–2009 in the USA [2,3,4]. Cutaneous melanoma mostly occurs in patients between 40 and 60 years old, and is the most common form of cancer in young adults 25–29 years old [5]. Men over 65 have a higher incidence of nodular melanoma, and their 10-year disease-specific survival being lower than for younger patients [6].

A variety of environmental, genetic, and social factors increases the risk for developing malignant melanoma [2,3]. Environmental risk factors include: acute sun exposure, especially at a young age, associated with blistering sunburns; prior therapy with psoralen and ultraviolet A radiation (PUVA) for cutaneous T cell lymphoma (CTCL) or psoriasis; or congenital or acquired immunosuppression.

Inherited traits increasing the risk of developing a melanoma include family history of melanoma and hereditary syndromes associated with atypical moles and dysplastic nevi, which are more likely than ordinary moles to become malignant [7]. Individual risk factors include: age, number of nevi, skin type and color (phototype), personal history of cutaneous melanoma or non-melanoma skin cancer (NMSC), freckles, dysplastic nevi, and fair hair color.

Eight percent of melanoma patients develop a secondary melanoma within 2 years of their initial diagnosis [8]. Melanoma patients also have increased risk of developing other NMSC [9]: 35% of the patients with lentigo maligna melanomas develop another cutaneous malignancy within 5 years [10].

Moreover, recent decades have witnessed an increased mobility of the northern populations toward southern tropical countries for short vacations. There is a general thought that tanned skin is a sign of a healthy look and of a wealthy status. Sunbed use spread among teenagers and young adults, although this habit has been shown to increase melanomas [11,12].

Although a meta-analysis failed to demonstrate a survival advantage for melanoma screening in large populations [13], there are a few convincing reports about the importance of early melanoma detection [14,15,16,17]. Between 2007 and 2011, compared to the period 2002–2006, the annual costs for treating skin cancers increased by 126%, from 3.6 to 8.1 billion USD, highlighting the potential importance of skin cancer prevention efforts in future savings for the healthcare system [18].

Early detection is considered effective in reducing melanoma mortality [19], but screening the whole population was found to be very expensive and not feasible [20]. Several screening studies have been conducted in different European countries in recent years [14,19,21] with contradictory results [20]. Since screening a population displaying melanoma risk factors (secondary prevention) is considered more cost effective than screening an unselected population (primary prevention), most guidelines recommend annual skin checks in individuals who display known melanoma risk factors [13,22]. Education about skin self-examination is widely encouraged together with avoiding the environmental risk factors and sunbed use.

However, there are inconsistencies in screening and follow-up recommendations for individuals with an increased risk of developing a melanoma [23,24]. Most guidelines recommend that these individuals should be monitored, but only half provide recommendations on a standardized recruiting method or for screening based on level of risk [25]. Although controversies are reported in the literature for melanoma screening as well as for breast or colon cancer prevention [26,27,28,29], melanoma screening is not supported by the Italian public health system, leaving the initiative to national dermatology societies or non-profit organizations. The Associazione Controil Melanoma OdV (ACM) was founded in 2004, focusing on melanoma screening and prevention, public education and information, melanoma patient care, and economic support of research projects. Currently, ACM has over 500 members, including volunteers physician, melanoma patients and relatives, and non-physician volunteers. ACM membership includes, as a benefit, yearly dermatologic skin examinations for each member and possibly first-degree relatives. ACM can count on the voluntary activity of 20 specialists either in dermatology (13) or plastic surgery (7).

With the present analysis, we aimed at defining which of three methods described below is most effective in selecting a high-risk population for successful melanoma screening as indicated by guidelines. Our aims included the quantification of the differences between three recruiting schemes to detect which, if any, best performs in selecting a population with melanoma risk factors to be effectively screened for melanoma.

## 2. Materials and Methods

Subjects were recruited among the ACM network, or among unselected population through public campaigns on local media, and in drug stores, following three different screening methods:

1. Regular skin examinations (RS), conducted all year around primarily within the ACM network, from January 2010 to December 2018;
2. Occasional short screening campaigns (OS) lasting 2 weeks every year, from January 2010 to June 2016;
3. Selective screening (SS) information campaigns held for 2 weeks yearly where persons were evaluated for the risk of developing melanoma through a quick questionnaire using 10 questions and adapted from the Australian Victorian Melanoma Service Questionnaire [30,31]. Those who fit an intermediate/high risk profile, according to the questionnaire, were recommended to undergo a screening examination from June 2016 to December 2018.

Skin examinations were performed by dermatologists or plastic surgeons. Traditionally, in the University Hospital of Pisa, either plastic surgeon or dermatologist specialists, are trained to develop expertise in early melanoma diagnosis.

**Figure 1:**
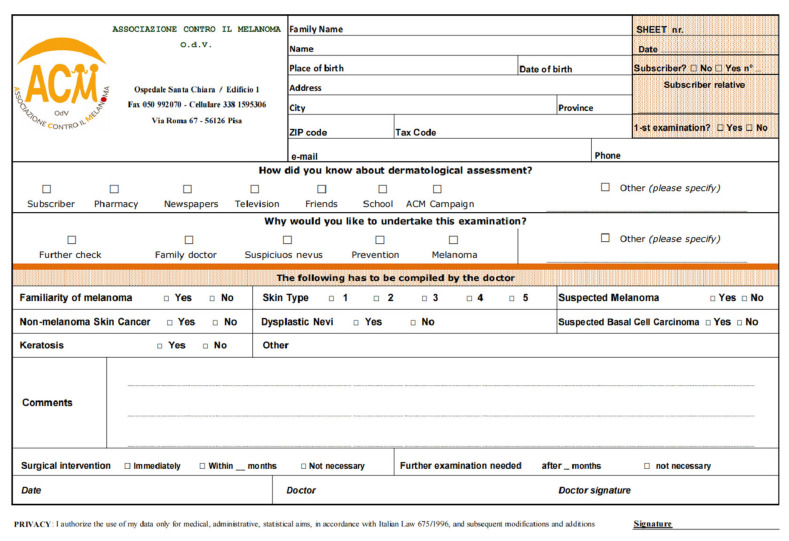
Form filled by subjects and doctors at each visit. O.d.V. (Organizzazione di Volontariato) is a kind of non-profit organization.

Skin examination appointments were scheduled in advance, and participants signed a disclaimer form, which was also notified to the local ethical committee. Skin examinations were conducted with the aid of a digital video dermatoscope. Subjects and doctors filled out a form at each visit (Figure 1).

A few questions were aimed at determining how the subjects learned about the campaign. Data collected from the forms were the source of the present analysis.

Clinical outcomes included: sex, age, family history of melanoma, personal history of melanoma, skin phototype, dysplastic nevi, NMSC, and seborrheic keratosis. Further information included location and date of visits (Figure 1).

Whenever excisions of suspicious lesions were deemed necessary, subjects were referred to the plastic surgical department of the University Hospital of Pisa. Surgical and pathology reports for all diagnostic and therapeutic procedures were then collected. The local ethics committee was notified of the protocol.

Only the first skin check for each subject was evaluated for the purpose of this analysis. We considered comparisons RS vs. OS, RS vs. SS, and SS vs. OS. Univariable analysis was performed using a chi-square test, applying Fisher’s exact test correction in cases of small sample sizes. Student’s *t* or Mann–Whitney tests were used to compare means. In a multivariable analysis, generalized linear mixed models (logit GLMMs) were fitted and used to test the significance of parameters reported in Table 2, using family-wise confidence interval estimates. The fixed effects were the four main risk factors: family history of melanoma, personal history of melanoma, skin phototype, and dysplastic nevi. Random effects were sex and age. Statistical analyses were performed using the oper source software R Project for Statistical Computing, version 4.0.1 (The R foundation, 06/06/2020), using a significance level of 0.05.

## 3. Results

Data were collected for the screening activity by ACM from January 2010 to December 2018, in 16 screening locations in Tuscany, mainly in the provinces of Pisa and Lucca, Italy. The characteristics of the population under study are illustrated in Table 1.

**Figure 2:**
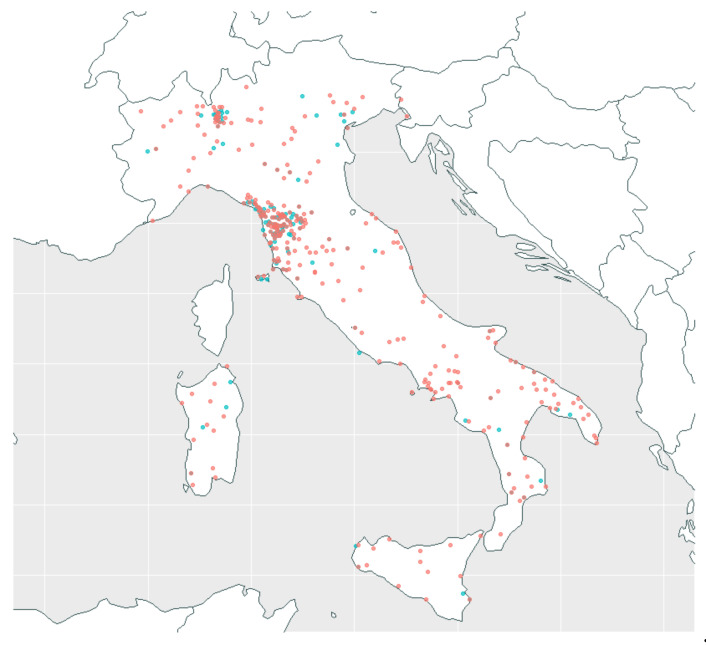
Map of municipalities of residence (207, in blue) and places of birth (407, in red) of screened subjects.

A total of 2238 subjects, from 207 different municipalities of residence and 407 places of birth throughout Italy (see Figure 2), were screened: 835 men and 1403 women. The average age was 43.18 years, with a standard deviation of 18.34. About 70% were between 25 and 65 years of age.

A total of 1390 subjects (62.1%) answered the question, “How did you learn about ACM?” as follows: in 13.5% of the cases, the information was spread by word of mouth; in 20.5% of the cases, by ACM subscribers or campaigns; in 5.9% of the cases, by local media; the remaining is represented by less frequent or not categorized options. Among the 1369 subjects that checked the item, 84.2% reported prevention as the main reason to undergo a skin check (Table 1).

Family history of melanoma, personal history of melanoma, skin phototype, and dysplastic nevi were the main recorded risk factors (Table 2). Of the whole population, 15% had a family history of melanoma (15.8% in RS, 12.2% in OS, and 19.2% in SS), and 3.3% had a personal history of melanoma (3.1% in RS, 3.9% in OS, and 2.2% in SS). High-risk and low-risk skin phototypes were quite uniformly represented. Seborrhoeic keratosis was the most frequently diagnosed skin lesion, present in 20.2% of cases, followed by dysplastic nevi (1.7%), NMSC (1.2%), and melanomas in two subjects.

Skin checks were performed by 20 doctors: 7 plastic surgeon specialists, who performed a total of 866 skin checks (average 123.7, range 23–353) and 13 dermatologists, who performed a total of 1085 skin checks (average 83.5, range 14–454).

Repeated skin checks based on clinical findings were recommended by the visiting doctors to 59.7% of subjects (Table 1). Doctors recommended surgical excision of suspicious lesions in 8.9% of cases. After 2 years of follow-up, 71.4% of subjects followed the doctor’s recommendation, while 28.6% declined. We collected 45 pathology reports, reporting 41 benign lesions, 2 basal cell carcinomas, and 2 melanomas. Pathology reports were not available in five cases because surgery was performed outside the network.

### Comparison among the Three Recruiting Methods

To evaluate which recruiting method produced the best yield of a population enriched for melanoma risk factors, we compared the frequency of melanoma risk factors across the three groups: OS, RS, and SS. The characteristics of the three groups are summarized in Table 2.

Age distribution differed between the three groups especially in ages 0–25 years and over 65 years, but showed a similar pattern in the central age groups. Overall, median ages differ between the groups: RS had a median age of 44 years, OS a median age of 43 years, and SS had a median age of 46 years (*p* < 0.00054). The percentage of women was higher in OS (66.4%) than in SS (59.7%) groups (*p* < 0.019) according to a chi-squared test.

Statistically significant differences in risk factors were observed only comparing RS to OS or SS to OS. The comparison between RS and SS did not lead to a statistically significant difference in risk factors. A family history of melanoma was reported more frequently in RS (15.8%) and SS (19.2%) than in OS (12.2%), the differences being statistically significant. Skin phototypes I-II were significantly more represented in RS (59.7%) and SS (65.7%) than in OS (47.9%). Personal history of melanoma and dysplastic nevi did not show any significant difference between RS and OS groups. Both recommended follow-up and removal of suspected lesions were more frequently advised among SS group (Table 2).

## 4. Discussion

Possible options for improving the early detection of melanoma include general population dermatological screening, targeted screening of a high-risk group, self-screening, and skin awareness. Future strategies to improve and maintain both a high level of early detection by the general public and accurate recognition of suspicious lesions by specialists need to be evaluated in terms of workload, psychological outcome, and economic costs. Efforts to encourage screening should include specific guidelines on the age at which skin cancer screening should begin and the frequency with which it should be repeated.

Due to the low incidence of melanoma in Italy (13.5 cases/year over 100,000 inhabitants) [32], for a general population screening to be cost-effective, the following requirements should be fulfilled: a good uptake rate, a practical and accurate method for identifying the target population, and a selection of a target population that ensures a high yield of melanoma diagnosis [25,33]. Thus far, no unique scoring system has been developed to discriminate high-risk vs. low-risk individuals with respect to melanoma development. Although a few melanoma scales are available, they have not been validated in a large population [34].

Ideally, a screening method should be validated by survival analysis, but that would require a very large randomized study. The scale we selected has the advantage of being simple, with few items and quick to fill in. All the international melanoma screening guidelines report the effectiveness of conducting skin checks in individuals harboring melanoma risk factors, but none of them indicate a strategy to effectively recruit those subjects [35].

This study reports the results of a melanoma screening activity organized and conducted with very limited resources by a patient non-profit organization. The findings show that either a pre-selection of subjects with the help of a risk evaluation questionnaire or regular skin examinations conducted within ACM network are more effective in recruiting a population enriched for melanoma risk factors.

The SS method performed better in recruiting a population enriched for melanoma risk factors compared to the RS method, although the difference was not statistically significant. Compared to OS, the SS method reached a statistically significant difference in the same aspect. SS method, being more selective, allows obtaining more information regarding risk factors compared to the other two methods of selection.

Subjects in the RS group were generally more aware of the main melanoma risk factors (family history for melanoma, previous melanoma diagnosis, and skin phototype I–II) and were more prone to follow the recommendation for regular skin checks. OS subjects, recruited in summer campaigns, adhered, driven by anxiety, having heard about risk factors mostly for the first time and/or allured by the possibility of having a skin check for free. Therefore, we consider this method, although useful to reach a wider part of the population, less suitable to enroll a higher-risk population.

SS subjects were not offered a skin check immediately, but they agreed to have their risk of developing a melanoma scored first, and only in case of intermediate/high risk were they offered a free skin check. Because of this, among SS, no subject driven only by anxiety was recruited and any worrisome question could be addressed by the ACM-trained volunteers who administered the questionnaire.

A total of 1 melanoma every 1119 people screened does not appear to be a very effective yield, but as both melanomas were diagnosed in the RS group, this represents 1 melanoma every 547 subjects screened and 1 every 43.5 excised lesions in this population group.

About 46% of the recruited population was represented by women aged 26–65 years, showing that we failed to recruit subjects at the highest risk of dying of melanoma [6], i.e., men over 65 years, representing only 5.5% in our population. In this respect, we plan to involve primary physicians inviting them to refer those subjects to our ambulatories by offering them a user-friendly computerized system to set the appointments [36].

The 17.9% recruitment of a population ≤25 years old is probably due to the long-term activity of ACM in educating children and young adults in primary and secondary schools on the advantages of skin protection and shade seeking all year around.

Recommended follow-up was more frequently suggested in OS than in RS, probably because OS was mainly represented by subjects at first recruited through occasional campaigns, to whom the specialists had pointed out one or more melanoma risk factor(s), therefore recommending periodic skin checks.

We found that SS better suits our purpose to select a population with higher frequency of two of the main recognized melanoma risk factors, i.e., phototype and family history of melanoma. Other methods should be implemented to recruit male subjects over 65 years, which represent a population affected by melanomas at high risk of mortality [6]. In the future, molecular biomarkers could more effectively select among the general population subjects at higher risk of developing a melanoma [37].

Based on these findings, ACM has extended SS all year around, to allow the possibility to evaluate the risk of developing a melanoma through a link in its web site (www.associazionecontromelanoma.it accessed on 7 October 2020) to the Victorian Melanoma Service Questionnaire [30], advising only subjects at intermediate and high risk to set an appointment with a dermatologist for regular skin checks. The effectiveness of such a method in early melanoma diagnosis will be evaluated in future studies.

## Figures and Tables

**Table 1:** Patient characteristics, including recommendations of periodic skin checks or surgical removal of suspicious lesions, source of information of the campaign activities, and reasons to undergo skin check. NA, missing data (not available).

Variable	Levels	Counts (%)
Age (years)	1–25	402 (18.6)
	26–45	742 (34.3)
	46–65	754 (34.9)
	66–85	264 (12.2)
	NA	76 (3.4)
Sex	F	1403 (62.7)
	M	835 (37.3)
	NA	0 (0)
Family history	NO	1903 (85.0)
	YES	335 (15.0)
	NA	0 (0)
Skin phototype	III-IV-V	1009 (45.1)
	I-II	1229 (54.9)
	NA	0 (0.0)
Melanoma history	NO	2165 (96.7)
	YES	73 (3.3)
	NA	0 (0.0)
Dysplastic nevi	NO	2199 (98.3)
	YES	39 (1.7)
	NA	0 (0.0)
Seborrheic keratosis	NO	1787 (79.8)
	YES	451 (20.2)
	NA	0 (0.0)
Non-melanoma skin cancer	NO	2212 (98.8)
	YES	26 (1.2)
	NA	0 (0.0)
Recommended follow-up	NO	1028 (45.9)
	YES	1179 (59.7)
	NA	31 (1.4)
Surgery indication	NO	2007 (89.7)
	YES	200 (8.9)
	NA	31 (1.4)
How did people learn about us?	Friends	303 (13.5)
	Pharmacists	77 (3.4)
	Media	133 (5.9)
	School	13 (0.6)
	ACM subscribers	300 (13.5)
	ACM campaigns	160 (7.1)
	Other	404 (18.1)
	NA	848 (37.9)
Reason for skin check	Presence of a Skin lesion	204 (9.1)
	Prevention	1153 (51.5)
	Other	12 (0.5)
	NA	869 (38.8)

**Table 2:** Frequencies of occurrence of clinical outcomes comparing RS, OS, and SS, with *p*-values indicating statistical significance of both univariable and multivariable models. Dash indicates the variable is not included in the model, while n.s. stands for not significant. NA, missing data (not available).

Variable	Counts (%)			*p*-Value (Univar.)	*p*-Value (Multivar.)
	RS1094 (48.9)	OS826 (36.9)	SS318 (14.2)		
**Age (years)**				6.479 × 10^−5^	-
≤25	218 (19.9)	139 (16.8)	45 (14.2)		
26–45	352 (32.2)	279 (33.9)	111 (34.9)		
46–65	354 (32.4)	291 (35.2)	109 (34.3)		
≥65	133 (12.2)	78 (9.4)	53 (16.7)		
NA	37 (3.4)	39 (4.7)	0 (0.0)		
**Sex**				0.02093	-
F	668 (60.9)	549 (66.4)	190 (59.7)		
M	428 (39.1)	277 (33.6)	128 (40.3)		
NA	0 (0.0)	0 (0.0)	0 (0.0)		
**Family history**				0.006994	0.0115
NO	921 (84.2)	725 (87.,8)	257 (80.8)		
YES	173 (15.8)	101 (12.2)	61 (19.2)		
NA	0 (0.0)	0 (0.0)	0 (0.0)		
**Phototype**				5.92 × 10^−15^	1 × 10^−5^
III-IV-V	438 (40.0)	462 (55.9)	109 (34.3)		
I-II	656 (60.0)	364 (44.1)	209 (65.7)		
NA	0 (0.0)	0 (0.0)	0 (0.0)		
**Melanoma history**				n.s.	n.s.
NO	1060 (96.9)	794 (96.1)	311 (97.8)		
YES	34 (3.1)	32 (3.9)	7 (2.2)		
NA	0 (0.0)	0 (0.0)	0 (0.0)		
**Dysplastic nevi**				n.s.	n.s.
NO	1078 (98.5)	810 (98.1)	311 (97.8)		
YES	16 (1.5)	16 (1.9)	7 (2.2)		
NA	0 (0.0)	0 (0.0)	0 (0.0)		
**Seborrheic keratosis**				n.s.	-
NO	855 (78.2)	674 (81.6)	258 (81.1)		
YES	239 (21.8)	152 (18.4)	60 (18.9)		
NA	0 (0.0)	0 (0.0)	0 (0.0)		
**Non-melanoma skin cancer**				n.s.	-
NO	1046 (95.6)	793 (96.0)	294 (92.5)		
YES	48 (4.4)	33 (4.0)	24 (7.5)		
NA	0 (0.0)	0 (0.0)	0 (0.0)		
**Recommended follow-up**				2.2 × 10^−16^	-
NO	565 (51.7)	407 (49.3)	56 (17.6)		
YES	507 (46.3)	413 (50.0)	259 (81.5)		
NA	22 (2.0)	6(0.7)	3(0.9)		
**Advised Surgical Removal**				0.04405	-
NO	985 (90.0)	747 (90.5)	275 (86.5)		
YES	87 (8.0)	73 (8.8)	40 (12.6)		
NA	22 (2.0)	6(0.7)	3(0.9)		
